# Fungal interactions induce changes in hyphal morphology and enzyme production

**DOI:** 10.1080/21501203.2021.1932627

**Published:** 2021-06-04

**Authors:** Samim Dullah, Dibya Jyoti Hazarika, Assma Parveen, Merilin Kakoti, Tanushree Borgohain, Trishnamoni Gautom, Ashok Bhattacharyya, Madhumita Barooah, Robin Chandra Boro

**Affiliations:** a Department of Agricultural Biotechnology, Assam Agricultural University, Jorhat, Assam, India; b Department of Plant Pathology, Assam Agricultural University, Jorhat, Assam, India; c Royal School of Bio-Sciences, Royal Global University, Guwahati, India

**Keywords:** Barrage formation, deadlock, fungal–fungal interaction, metabolite, scanning electron microscopy, *Trametes*

## Abstract

In nature, species interacts/competes with one other within their surrounding for food and space and the type of interactions are unique to each species. The interacting partners secrete different metabolites, which may have high importance in human welfare. Fungal–fungal interactions are complex mechanisms that need better understanding. Here, 14 fungal isolates were facilitated in 105 possible combinations to interact on potato dextrose agar. Morphologically, no changes were observed when the same fungal isolates were allowed to interact within them. However, 10 interactions between different fungal isolates showed mutual replacement with each fungus; capturing territory from the other. Contrastingly, 35 interactions resulted into complete replacement as one of the fungi was inhibited by rapid growth of the other fungus. In 46 interactions, formation of barrage was observed leading to deadlock type of interaction wherein both fungi have restricted growth. To study in details about the barrage formation, two fungal interactions were taken (i) *T. coccinea* vs. *L. lactinea* and (ii) *T. coccinea vs. T. versicolor*. Microscopic changes in the hyphal growth during interaction were observed. There was significant increase in the enzymatic activities including cellulase, xylanase and chitinase during *in-vitro* fungal–fungal interaction, suggesting the importance of such interactions for commercial enzyme production.

## Introduction

Fungi are achlorophyllous, cryptogamous life forms that are fundamental biotic component of all major ecosystems and play vital role as decomposers that aid in nutrient recycling (Peay et al. 2008). Fungi usually live and grow close to each other in their natural environment; they also interact with the other microbes living together in mixed populations with complex interactions like symbiosis and competition. Colony relations within fungal groups are reported to have effect on mycelial spreading, resulting in interactions which become indeterminate and are ubiquitous. Factors contributing to fungal community growth in specific biotic and abiotic environment have been reported (Falconer et al. 2008). The interaction of fungus leads to several consequences such as intermingling/neutral interaction which results in fusion of colonies for compatible or spatial intermixing for incompatible genotypes. Interaction may also lead to a deadlock, with neither of the species entering the territory of the other; or replacement, where one individual is partially or entirely replaced by another (Boddy 2000). During deadlock, strong competition occurs between the two interacting fungal species.

For many of the fungal groups, wood is a primary habitat (Rajala et al. 2015). The wood inhabiting basidiomycetes, which constitute the polypore fungi as one of the groups, are mostly responsible for wood decomposition. These fungi can degrade both cellulose and hemicelluloses along with lignin. They grow in close proximity to each other, resulting in a series of interaction between them in the form of competition and synergistic activities (Song et al. 2012). Dual culture between various wood rotting fungi has been extensively studied to decipher the characteristic changes in the extracellular enzyme activities during the interaction process as for example, superoxide dismutase involved in detoxification, 1,3 glucan synthase involved in nutrient acquisition and growth, ubiquitin involved in protein metabolism (Chi et al. 2007). In our study, we aimed at understanding the changes that occur in both morphological as well as in the metabolic activities particularly giving emphasis to the industrially important hydrolytic enzymes - xylanase and cellulase – during interaction between fungal isolates in the *in-vitro* conditions.

## Materials and methods

### Organisms and culture conditions

Fourteen fungal isolates previously isolated from different wood and soil samples; available in the department of Microbial Biotechnology Laboratory, Department of Agricultural Biotechnology, Assam Agricultural University, Jorhat were used in this study. The fungal isolates were inoculated on Potato Dextrose Agar (PDA) following standard protocol. The pure cultures were regularly sub-cultured at every 3 weeks and maintained at 28°C.

### DNA extraction, PCR and sequence analysis

Total DNA from each of the 14 fungal isolates were extracted and polymerase chain reaction (PCR) amplification was performed using a universal primer targeting the Internal transcribed spacer (ITS) {ITS1 (5ʹ-TCCTCCGCTTATTGATATGC-3ʹ) and ITS4 (5ʹ-TCCTCCGCTTATTGATATGC-3ʹ)}, following the method described by Parveen et al. (2017). The amplified products were sequenced by Bioserve Biotechnology, India. The sequence results thus obtained were compared to those available in the GenBank using BLASTn and submitted to GenBank. Phylogeny tree was constructed using MEGA 6.0 software by neighbour joining method with Kimura-2 parameter (K2P) correction and 1000 bootstrap replications. The K2P model was selected by model prediction in MEGA 6.0 considering the fact that models with the lowest BIC scores (Bayesian Information Criterion) are considered to describe the substitution pattern the best.

### Assessment of interactions by dual culture method in culture media

Possible combination interactions of the fungal samples were analysed by dual-culture technique. For each interaction, there were two different control plates containing each of the fungi inoculated on one half and the other half was kept uninoculated. The cultures were incubated at 28°C and regularly observed for 14 days. The inference on the type of interaction was made on the 14th day post inoculation following the method described by Skidmore and Dickinson (1976) especially the behaviour of the fungi in the zone of interaction.

### Light microscopic visualisation of interacting fungi

The interaction between *T. coccinea* – *T. versicolor* and *T. coccinea* – *L. lactinea*, showed inhibition with a narrow demarcation line. Microscopic observations were performed in three different ways, namely (1) Interaction of fungi on clean grease-free glass slides containing 1% water agar media. Control slides contain single culture and were incubated at 28°C. The cultures were observed under the light microscope (Olympus BX51, Olympus Corporation, Japan) after 3 days post inoculation by staining with lactophenol cotton blue (Himedia, India). (2) Mono-cultures were interacted with the metabolic extracts of *T. coccinea, T. versicolor* and *L. lactinea*, whereby the metabolites were extracted from the hyphae of each of the strains by incubating with methanol:dichloromethane:ethyl acetate (1:2:3) for 12 h and then sonicated. The extracts obtained were filtered and dried in a vacuum evaporator and resuspended in 2 ml of Dimethyl Sulphoxide (DMSO) and filtered through 0.22-µm syringe filter (Whatman, USA). Each strain was grown in 1% water agar slides against the different metabolic extracts followed by microscopic observation. (3) Interaction of mono-culture with extracellular component – of *T. coccinea, T. versicolor* and *L. lactinea* grown in liquid medium containing Potato Dextrose Broth (PDB) for 8 days. Culture supernatant was collected and filtered through 0.22 µm syringe filter. Each isolate was allowed to grow in 1% water agar slides and 50 µl of supernatant was added on the water agar surface. Hyphae were allowed to grow and then observed under the microscope. Controls were maintained for each interaction.

### Visualisation of interacting fungi under scanning electron microscopic (SEM)

The cultures of *T. coccinea* vs. *T. versicolor* and *T. coccinea* vs. *L. lactinea* were grown in PDA plates and the hyphae from both of the interacting cultures and as well as from the control cultures were taken and fixed in 2.5% glutaraldehyde (prepared in 0.1 M phosphate buffer). Sample preparation for SEM analysis was done following the protocol of Kathuria et al. (2014). SEM analysis was done using FEI Quanta 250 SEM at an accelerating voltage of 10 kV with a scanning electron detector for taking micrographs at different magnifications.

### Qualitative and quantitative screening of xylanase enzyme

Fungal xylanase screening was done following method described by Salmon et al. (2014) with slight modifications. The isolates *T. coccinea, T. versicolor* and *L. lactinea* were inoculated in separate flask containing xylan broth medium (XBM) and incubated at 28°C for 8 days. Supernatant from each of the cultures were extracted by centrifuging at 12,000 rpm for 10 min. The supernatant was added in the wells of the media supplemented with 1% Birchwood xylan as substrate in the petriplates. The plates were then incubated at 25°C for 3 days. After that, plates were rinsed with 0.5% Congo red for 15 min and then rinsed with 1 M NaCl for 15 min. Clear zone around the well in the Petri plates was considered to be positive for xylanase enzyme. Quantitative test was done following the method as described by Irfan et al. (2016). The reaction mixture comprised 0.9 ml of 50 mM Sodium acetate buffer (pH-5.3), 0.5 ml of 1% xylan, 0.1 ml crude enzyme extract and was incubated for 50°C for 15 min. The reaction was stopped by adding 1.5 ml of dinitrosalicylic acid (DNS) and again incubated at 90°C for 10 min. Reduction of DNS was monitored spectrophotometrically by measuring the increase in absorbance at 550 nm against a reagent blank (reaction mixer without enzyme) and enzyme control (reaction mixer with boiled enzyme). One unit of enzyme activity was defined as the amount of enzyme required to produce 1 µmole reducing sugar as a xylose equivalent per minute under standard assay conditions. Spectrophotometric reading was taken for the mono and dual cultures at every 2 days for 14 days of post inoculation at an absorbance value of 550 nm.

### Qualitative and quantitative screening for cellulase

Khokhar et al. (2012) method was followed for qualitative detection of cellulase with slight modification. Isolates were inoculated in carboxymethyl cellulose broth (CMC) for 8 days and the supernatant was filtered using a 0.22-µm syringe. The filtrate was poured into the wells made in the CMC media petri plates. The plates were flooded with 1% Congo red solution and allowed to sit for 20 min at room temperature. The plates were then de-stained with 1 M sodium chloride solution. A clear zone around the wells depicted cellulase positive against dark red backgrounds. For quantitative determination of cellulose, the method described by Florencio et al. (2012) followed with slight modification. The assay reaction was carried out in a cuvette, containing 0.9 ml of 50 mM Sodium acetate buffer (pH-5.3), 0.5 ml of 1% carboxymethylcellulose sodium salt (CMC), 0.1 ml crude enzyme extract incubated at 37°C for 60 min. The reaction was stopped by adding 1.5 ml DNS and again incubated at 90°C for 5 min. Reduction of DNS was monitored spectrophotometrically at 540 nm against a reagent blank (reaction mixer without enzyme) and enzyme control (reaction mixer with boiled enzyme) prepared under similar conditions. One unit of enzyme activity was defined as the amount of enzyme which released 1 µmole of reducing sugars (expressed as glucose equivalent), per min of reaction. Spectrophotometric reading was taken for the mono and dual cultures from 2nd to 14th day post inoculation at an absorbance of 540 nm at every 2 alternative days.

### Qualitative and quantitative screening for chitinase

The method of Agrawal and Kotasthane (2012) was followed for qualitative detection of chitinase with slight modification. Isolates were inoculated in colloidal chitin broth for 8 days and the supernatant was filtered using a 0.22-µm syringe. The filtrate was poured into the wells made in the solid media petri plates supplemented with colloidal chitin. The plates were allowed to sit for 30 min at room temperature. Formation of purple colour zone around the wells depicted chitinase positive. For quantitative determination of chitinase, the method described by Agrawal and Kotasthane (2012) was followed with slight modification. The assay reaction was carried out in a cuvette, containing 0.3 ml of 1 M sodium acetate buffer (pH-4.6), 0.2 ml of 1% colloidal chitin, 1 ml crude enzyme extract incubated at 40°C for 60 min. The reaction was stopped by adding 0.25 ml DNS and again incubated at 90°C for 5 min. Reduction of DNS was monitored spectrophotometrically at 582 nm against a reagent blank (reaction mixer without enzyme) and enzyme control (reaction mixer with boiled enzyme) prepared under similar conditions. One unit of enzyme activity was defined as the amount of enzyme which released 1 µmole of reducing sugars (expressed as *N*-acetyl-β-d-glucosamine equivalent), per min of reaction. Spectrophotometric reading was taken for the mono and dual cultures from 2nd to 14th day post inoculation at an absorbance of 582 nm at every 2 alternative days.

### Statistical analysis

All data obtained from xylanase and cellulase assays were analysed in SPSS 25.0 software by one-way Analysis of Variance (ANOVA). Statistical Means were separated by Duncan multiple range (DMRT) at 5% probability level and where necessary, graphs were plotted in Microsoft excel.

## Results

### Molecular characterisation and phylogenetic analysis of the fungal isolates

**Figure 1.**
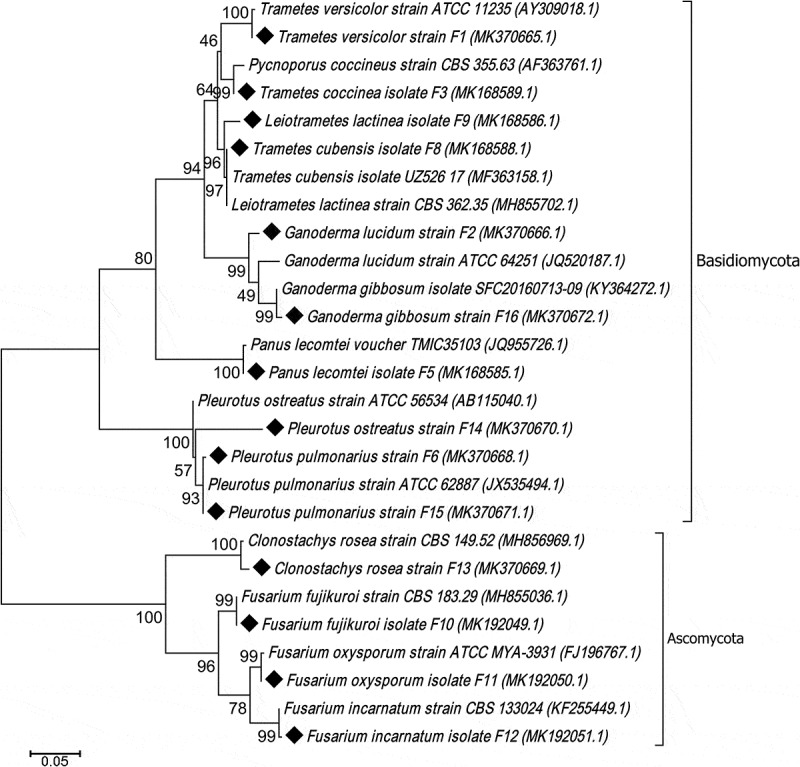
Phylogenetic tree of the 14 fungal isolates

Sequencing of the ITS region of the fungal DNA revealed the taxonomic identities of the 14 fungal isolates. BLAST analysis of the sequences revealed 97–99% homology with their respective reference sequences available in GenBank database of NCBI. Out of the 14 isolates, 10 isolates were identified as different species [*Ganoderma gibbosum* F16, *G. lucidum* F2, *Leiotrametes lactinea* F9 (synonym. *Trametes lectinea), Panus lacomtei* F5, *Pleurotus ostreatus* F14, *P. pulmonarius* F6, *P. pulmonarius* F15, *Trametes coccinea* F3 (synonym. *Pycnoporus coccineus), T. cubensis* F8, *T. versicolor* F1] of the phylum Basidiomycota. The other 4 isolates of the phylum Ascomycota were identified as *Clonostachys rosea* F13, *Fusarium fujikoroi* F10, *F. incarnatum* F12, *F. oxysporum* F11. The sequence information of the isolated was deposited to National Centre of Biotechnology Information (NCBI) database (Table 1). Phylogenetic analysis revealed that the isolates with close phylogenetic distances were clustered together, whereas distantly related isolates formed different clades (Figure 1). It was observed that most of the isolates were grouped with their reference isolates and there were species specific cluster formation in most cases, suggesting the effectiveness of ITS marker region for identification of fungi. However, the *Leiotrametes lactinea* and *Trametes cubensis* were grouped together (Figure 1) which signified that there were no sufficient differences in the sequences to determine the phylogenetic distances among these two species.

**Table 1.** The isolates used in the study along with the accession number

Isolate	Strain	Accession number
*Trametes versicolor*	F1	MK370665
*Ganoderma lucidum*	F2	MK370666
*Trametes coccinea*	F3	MK168589
*Panus lacomtei*	F5	MK168585
*Pleurotus pulmonarius*	F6	MK370668
*Trametes cubensis*	F8	MK168588
*Leiotrametes lactinea*	F9	MK168586
*Fusarium fujikoroi*	F10	MK192049
*Fusarium oxysporum*	F11	MK192050
*Fusarium incarnatum*	F12	MK192051
*Clonostachys rosea*	F13	MK370669
*Pleurotus ostreatus*	F14	MK370670
*Pleurotus pulmonarius*	F15	MK370671
*Ganoderma gibbosum*	F16	MK370672

### *Dual culture study during* in-vitro *fungal–fungal interaction*

**Figure 2.**
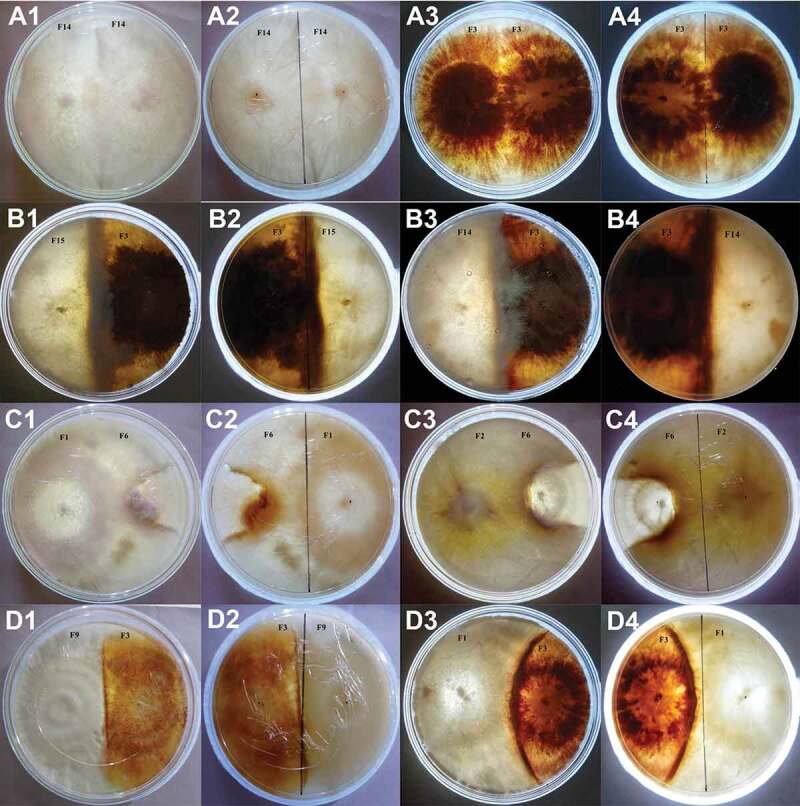
Different grades of interaction. **A1–A4** mutual intermingling growth without any macroscopic signs of interaction when the same strain is dual cultured. **A1** and **A2** front and back view during dual culture of *P. ostreatus*. **A3** and **A4** front and back view during dual culture of *T. coccinea*. **B1–B4** grade 2 type of interaction where the two fungal species grew either above or below each other. **B1** and **B2** front and back view of interaction between *P. pulmonarius* and *T. coccinea*. **B3**and **B4** front and back view of interaction between *P. ostreatus* and *T. coccinea*. **C1–C4** depict grade 3 type of interaction. **C1** and **C2** front and back view during the dual culture of *T. versicolor* with *P. pulmonarius*. **C3** and **C4** front and back view during the dual culture of *G. lucidum* with *P. pulmonarius*. In both the cases the growth of *P. pulmonarius* is ceased and is overgrown by the antagonistic fungi. **D1–D4** represent grade 4 type of interaction. **D1** and **D2** front and back view during the dual culture of *L. lactinea* and *T. coccinea*. **D3** and **D4** front and back view during the dual culture of *T. versicolor* with *T. coccinea*. In both the interactions the two fungi compete with one another. A demarcation line in the form of barrage zone is formed between them

In all, a total of 105 possible interactions between the fungal isolates were performed following the model of Skidmore and Dickinson (1976). Changes in mycelial morphology were most prominent in areas which were in direct contact with the competitor. When the mycelia of same fungal isolate were allowed to interact in dual culture, mutual intermingling between the hyphae occurred without any macroscopic signs of changes in hyphal morphology, which is considered as grade 1 type of interaction. Mycelia of fungal isolates in the same mycelial compatibility group showed the compatible reaction of intermingling and formation of a ridge of mycelia, for example, dual culture of *G. lucidum* with *G. lucidum* and *T. coccinea* with *T. coccinea* (Figure 2A).

Mutual replacement was observed, wherein, both the fungi capture territory from each other. These types of interaction were regarded as grade 2 type and 16 interactions in our study were found to be of this type, for example, interaction of *T. coccinea* with *P. ostreatus* and *T. coccinea* with *P. pulmonarius* (Figure 2B). In grade 3 type of interaction, complete replacement occurred, wherein one fungus displaces the other. In this study, 31 interactions were categorised as grade 3, as for example, interaction of *G. lucidum* with *P. pulmonarius* and *T. versicolor* with *P. pulmonarius* (Figure 2C). It was observed that the growth of the fungus is ceased and is being overgrown by the antagonistic fungus. In grade 4 type of interaction, two fungi in close proximity showed a characteristic barrage, light amber colour of the PDA media as well as the mycelia of both interacting partner changes to dark brown colour. Forty-three interactions were classified under this category, where both the interacting fungi show slight inhibition with a narrow demarcation line. This type of interaction thus resulted in deadlock where neither fungus loses their territory, for example, interaction of *T. coccinea* with *L. lactinea* and *T. coccinea* with *T. versicolor* (Figure 2D).

### *Light microscopic analysis during* in-vitro *fungal–fungal interaction*

**Figure 3.**
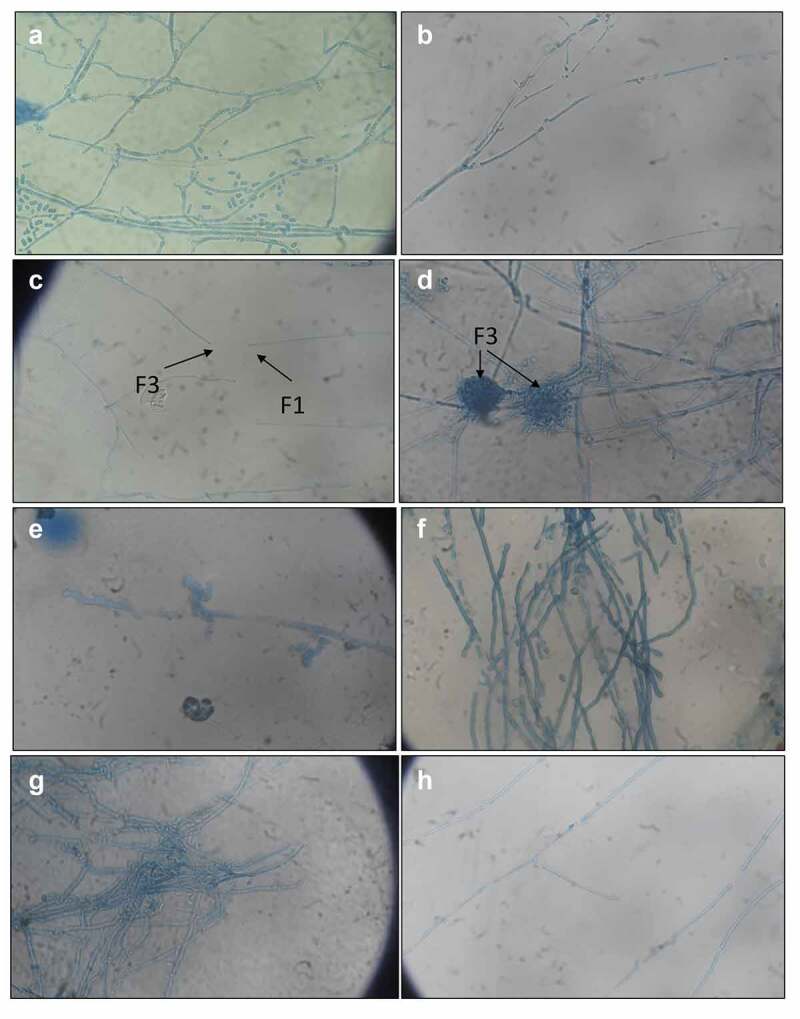
Microscopic analysis during interaction between *T. coccinea* and *T. versicolor*. (**A** and **B)** Hyphae of *T. coccinea* and *T. versicolor* respectively. (**C)** Hyphae of the two different fungal species approach towards each other. (**D)** shows the excessive sporulation caused by *T. coccinea* during the later stage of interaction. (**E)** depicts the distortion of the hyphae of *T. versicolor* when interacted with the metabolic extract of *T. coccinea*. (**F)** No change in hyphal structure when the hyphae of *T. coccinea* are interacted with the metabolic extract of *T. versicolor*. (**G)** Excessive branching of *T. coccinea* when interacted with the extracellular component of *T. versicolor*. (**H)** Dense mycelial network of the *T. versicolor* hyphae when interacted with the extracellular component of *T. coccinea.*

The fungal interactions studied under the light microscope between *T. coccinea* and *T. versicolor*, revealed that the two different hyphal strands during interaction resulted in the formation of a massive hyphal network with excessive sporulation in the interaction zone (Figure 3C & 3D), which was otherwise not observed in the hyphal pattern of the pure cultures (Figure 3A & 3B). Further microscopic observation revealed that the hyphae of *T. versicolor* were distorted (Figure 3E) when applied with the metabolic extract of *T. coccinea*. Also, the interaction of the hyphae of *T. versicolor* with the extracellular component of *T. coccinea* led to formation of dense mycelial network (Figure 3G), which is different from the normal branching pattern of *T. versicolor* (Figure 3B). Furthermore, when the hyphae of *T. coccinea* were interacted with the metabolic extract of *T. versicolor*, no change was observed (Figure 3F). Excessive branching was observed during the confrontation of the hyphae of *T. coccinea* with the extracellular component of *T. versicolor* (Figure 3H).

**Figure 4.**
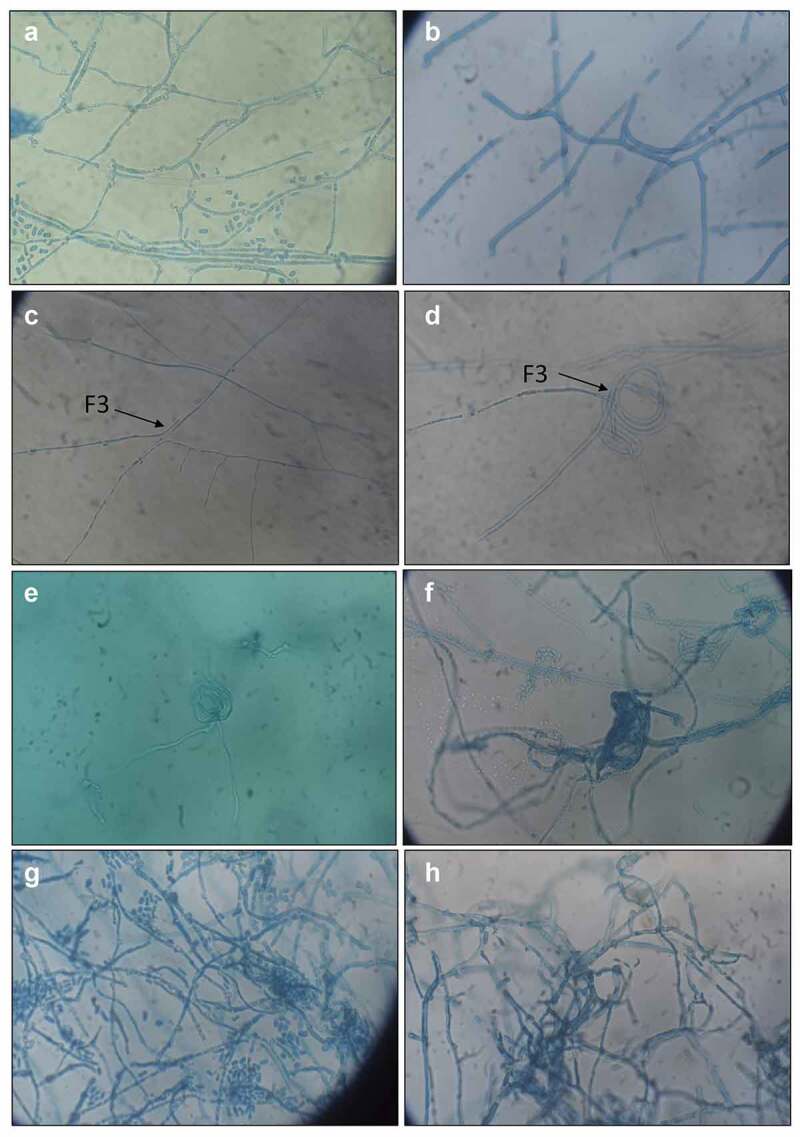
Microscopic analysis during interaction between *T. coccinea* and *L. lactinea*. (**A** and **B)** Hyphae of *T. coccinea* and *L. lactinea* respectively. (**C)** Hyphae of the two different fungal species approach towards each other. (**D)** Coiling of the hyphae of the two different fungal isolates during the later stage of interaction. (**E)** Coiling of the hyphae of *L. lactinea* when interacted with the metabolic extract of *T. coccinea*. (**F)** Coiling of the *T. coccinea* hyphae when interacted with the metabolic extract of *L. lactinea*. (**G)** Excessive sporulation of *T. coccinea* when interacted with the extracellular component of *L. lactinea*. (**H)** Irregular branching of the *L. lactinea* hyphae when interacted with the extracellular component of *T. coccinea.*

When *T. coccinea* was dual cultured with *L. lactinea*, coiling of the hyphae of *L. lactinea* was observed (Figure 4C & 4D). Interaction of the hyphae of *L. lactinea* with the metabolic extract of *T. coccinea* also resulted in hyphal coiling (Figure 4E). Similarly, treatment of the hyphae of *T. coccinea* with the *L. lactinea* metabolic extract induced hyphal coiling of *T. coccinea* (Figure 4F) and formed a dense network of mycelia due to excessive branching. The treatment of the hyphae of *T. coccinea* with the *L. lactinea* extracellular component resulted in excessive sporulation (Figure 4G) as compared to the control (Figure 4A). But the treatment of *L. lactinea* hyphae with the *T. coccinea* extracellular component resulted in irregular hyphal branching (Figure 4H) which otherwise is not observed in the control (Figure 4B)

### *SEM analysis during* in-vitro *fungal–fungal interaction*

**Figure 5.**
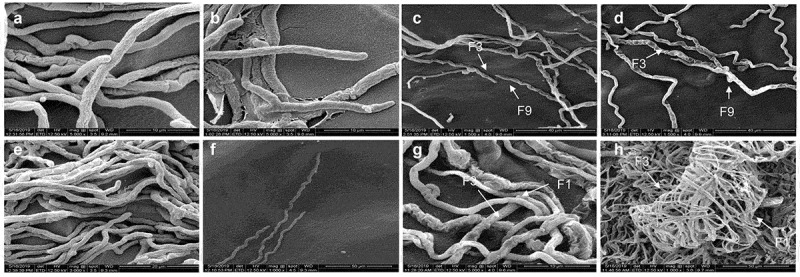
SEM analysis during *in-vitro* interaction. (**A** and **B)** Pure cultures of *T. coccinea* and *L. lactinea*. (**C)** Dense branching pattern between *T. coccinea* and *L. lactinea* when the two hyphae come close to each other. (**D)** Coiling like phenomenon observed during the interaction of *T. coccinea* with *L. lactinea*. (**E** and **F)** Pure cultures of *T. coccinea* and *T. versicolor*. (**G and H)** represents the massive network of hyphae during the interaction of *T. coccinea* and *T. versicolor* in low and high magnifications, respectively

The SEM analysis for the interaction between *T. coccinea* and *L. lactinea* initially led to dense branching pattern which later resulted in formation of coils in the interacting portion (Figure 5C & 5D) which was not observed in the pure cultures (Figure 5A & 5B). The interaction between *T. coccinea* and *T. versicolor* revealed formation of massive network of hyphae by both the interacting fungi (Figure 5G & 5H) as compared to pure cultures (Figure 5E & 5F).

### *Assessment of xylanase activity during* in-vitro *fungal–fungal interaction*

**Figure 6.**
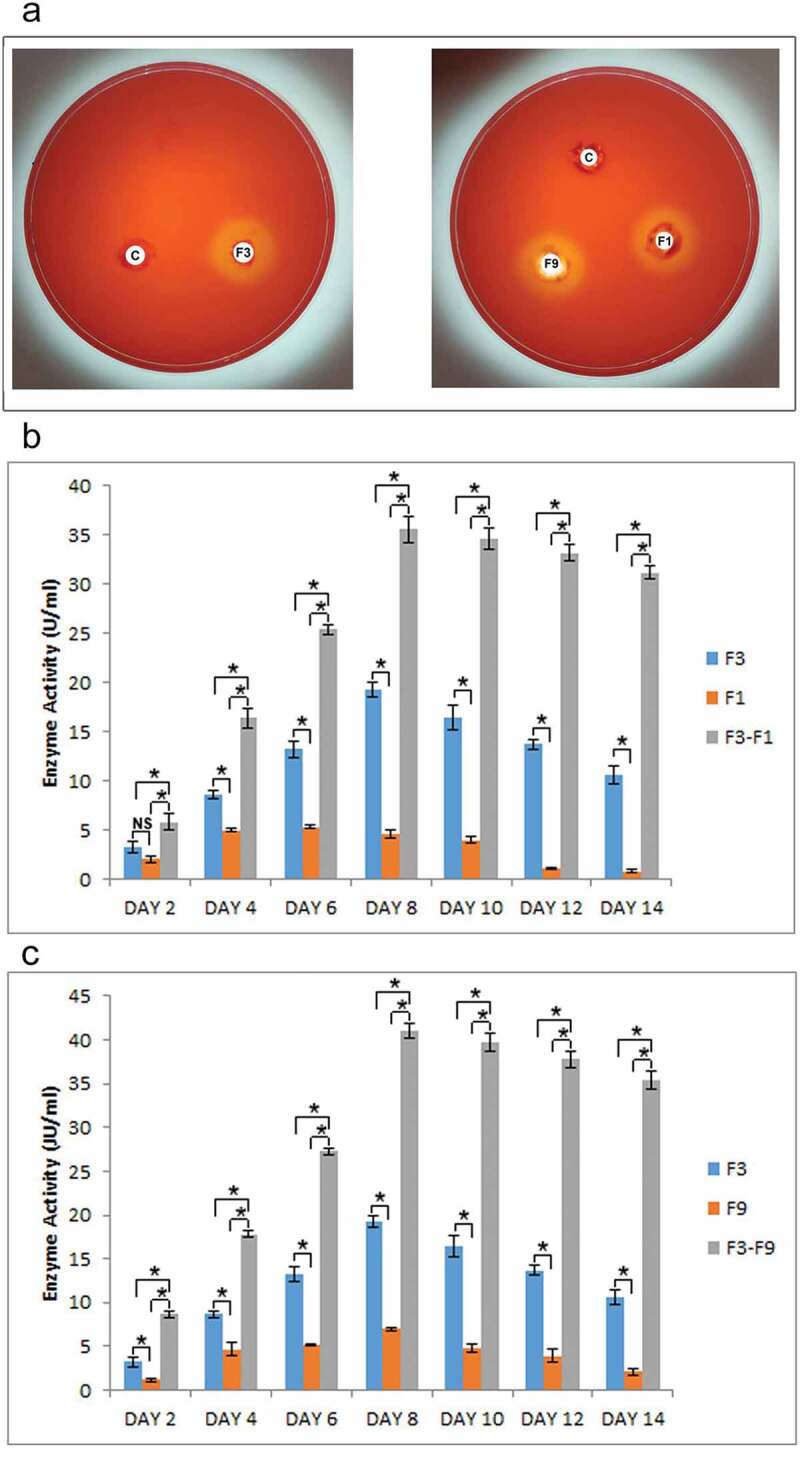
Xylanase activity. (**a)** The positive qualitative test for the *T. coccinea* (F3), *T. versicolor* (F1) and *L. lactinea* (F9) plate cultures. (**b)** Xylanase production by *T. coccinea* (F3), *T. versicolor* (F1) and their interaction (F3-F1). (**c)** Xylanase production by *T. coccinea* (F3), *L. lactinea* (F9) and their interaction (F3-F9). Asterisk (*) indicates level of significance with *p* ≤ 0.05. The *x*-axis represents days and *y*-axis represents the enzyme activity in U/mL

The plate assay for xylanase activity showed that *T. coccinea, T. versicolor* and *L. lactinea* were xylanase positive (Figure 6a). Quantification of xylanase activity revealed that the activity increased in the interactions compared to that in the pure cultures. The xylanase activity increased from 2nd day post inoculation up to the 8th day, and then slightly decreased on the succeeding days until the 14th day post inoculation for both the interactions (Figure 6b & 6c). The xylanase activity in case of the pure cultures also increased till the 8th days post inoculation but was less, as compared to the interaction ones. During, interaction the xylanase activity was higher, i.e. 35.5 U/mL for F3-F1 and 41.05 U/mL for F3-F9 as compared to the xylanase activity of the monocultures of *T. coccinea, T. versicolor* and *L. lactinea* was found to be 19.24, 4.59 and 6.97 U/mL, respectively.

### *Assessment of cellulase activity during* in-vitro *fungal–fungal interaction*

**Figure 7.**
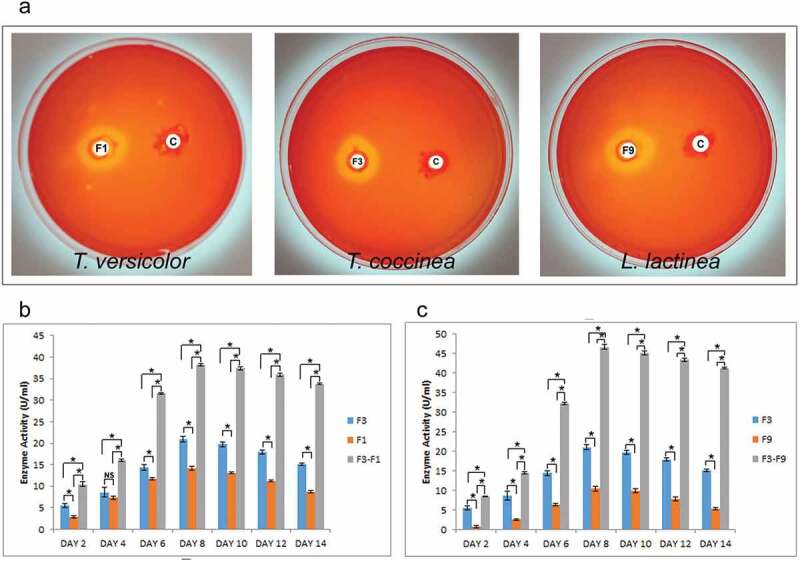
Cellulase activity. (**a)** depicts the positive qualitative test for the *T. coccinea* (F3), *T. versicolor* (F1) and *L. lactinea* (F9) plate cultures. (**b)** Cellulase production by *T. coccinea* (F3), *T. versicolor* (F1) and their interaction (F3-F1). (**c)** Cellulase production by *T. coccinea* (F3), *L. lactinea* (F9) and their interaction (F3-F9). Asterisk (*) indicates level of significance with *p* ≤ 0.05. The *x*-axis represents days and *y*-axis represents the enzyme activity in U/mL

The plate assay for cellulase activity showed that *T. coccinea, T. versicolor* and *L. lactinea* were cellulase positive (Figure 7a). Quantification of cellulase activity revealed that the interactions increased cellulase activities as compared to the enzyme activity in pure cultures. The cellulase activity increased from 2nd day post inoculation up to the 8th day, and then slightly decreased on the succeeding days until the 14th day for both the interactions (Figure 7b & 7c). The cellulase activity in case of the pure cultures also increased till the 8th day but was less, compared to the interaction ones. The cellulase activity for *T. coccinea* with *T. versicolor* (F3-F1) and *T. coccinea* with *L. lactinea* (F3-F9) was found to be 38.14 and 46.68 U/mL, respectively. This was more as compared to the cellulase activity of the monocultures of *T. coccinea, T. versicolor* and *L. lactinea* which were found to be 21.00, 14.24 and 10.46 U/mL, respectively.

### *Assessment of chitinase activity during* in-vitro *fungal–fungal interaction*

**Figure 8.**
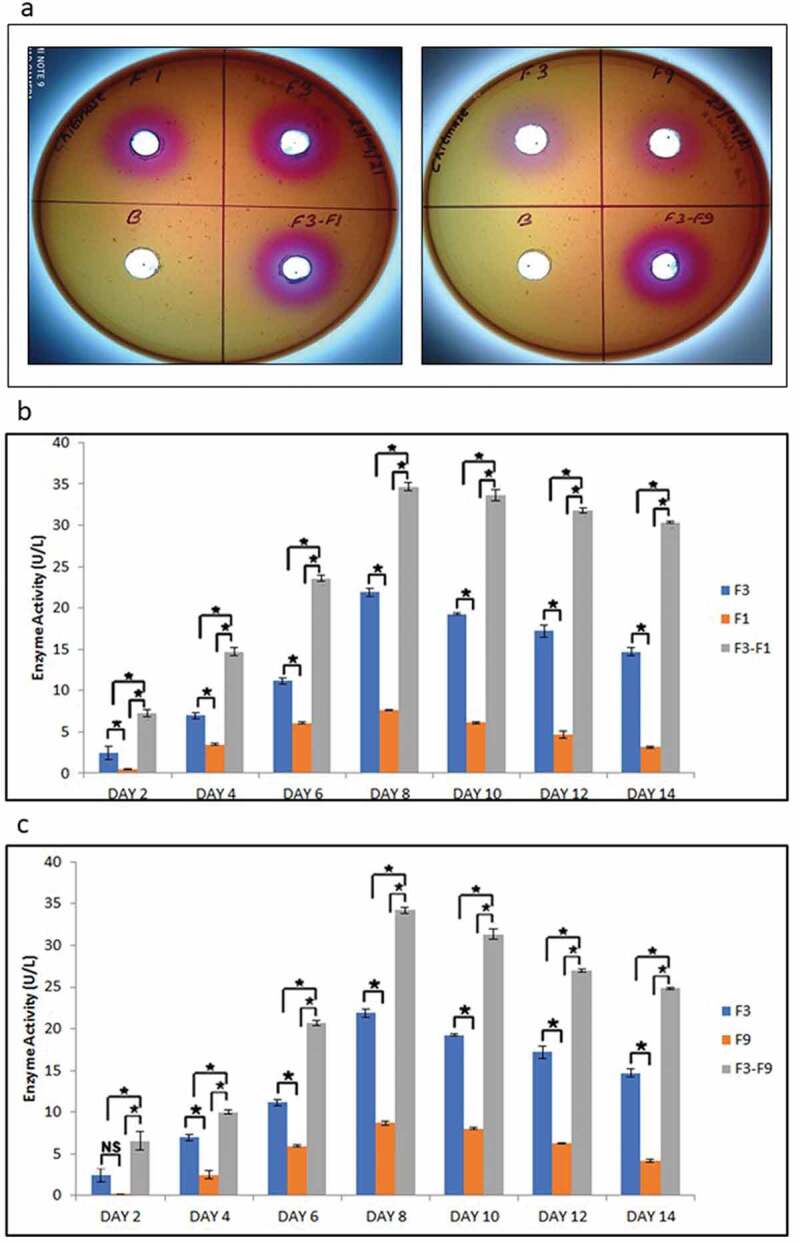
Chitinase activity. (a) depicts the positive qualitative test for the *T. coccinea* (F3), *T. versicolor* (F1) and *L. lactinea* (F9) plate cultures. (b) Chitinase production by *T. coccinea* (F3), *T. versicolor* (F1) and their interaction (F3-F1). (c) Chitinase production by *T. coccinea* (F3), *L. lactinea* (F9) and their interaction (F3-F9). Asterisk (*) indicates level of significance with *p* ≤ 0.05. The *x*-axis represents days and *y*-axis represents the enzyme activity in U/mL

The plate assay for chitinase activity showed that *T. coccinea, T. versicolor* and *L. lactinea* were chitinase positive (Figure 8a). Quantification of chitinase activity revealed that the interactions increased chitinase activities as compared to the enzyme activity in pure cultures. The chitinase activity increased from 2nd day post inoculation up to the 8th day, and then slightly decreased on the succeeding days until the 14th day for both the interactions (Figure 8b & 8c). The chitinase activity in case of the pure cultures also increased till the 8th day but was less, compared to the interaction ones. The chitinase activity for *T. coccinea* with *T. versicolor* (F3-F1) and *T. coccinea* with *L. lactinea* (F3-F9) was found to be 34.68 U/mL and 34.21 U/mL, respectively. This was more as compared to the chitinase activity of the monocultures of *T. coccinea, T. versicolor* and *L. lactinea* which were found to be 21.91, 7.67 and 8.67 U/mL, respectively.

## Discussion

Wood inhabiting and rotting fungi belonging mostly to the basidiomycetes group are very common in nature. Basidiomycetes and ascomycetes fungi produce important metabolite and enzymes that find use in white biotechnology. Studies have been carried out for fungal–fungal interaction between basidiomycetes, primarily aimed in profiling the changes in metabolite synthesis along with changes in enzymatic activities and gene expression (Yao et al. 2016; Xu et al. 2018; Zhong et al. 2018). In this study, unlike the previous studies from different authors, we demonstrated the physiological behaviour of the interacting hyphae through microscopy along with the change in enzyme activities when two different basidiomycetes fungi are dual cultured. Interaction of the 14 fungal isolates in all possible combinations among the basidiomycetes and ascomycetes fungus helped us to macroscopically observe the different types of fungal–fungal interactions (Table 2). In this study, we observed that most of the *in-vitro* interactions among and between white rot as well as brown rot fungi either led to complete replacement (grade 3) or deadlock (grade 4). Similar observations during the interaction process have been inferred by Owens et al. (1994). Grade 5 type of interaction where the hyphal growth of the two confronting fungi inhibits at a distance without any contact has not been observed in the study. From this observation, we can say that mutual inhibition at a distance is possible in case of interaction between lower groups of fungi. Similar results were observed by Mohammad et al. (2011) during dual-culture interactions between *Penicillium* sp. and *Trichoderma reesei* or can be a likely outcome during interaction of a basidiomycetes with lower fungal group such as *Penicillium* sp. Our primary focus was to study the deadlock interactions where strong competition occurs between the two interacting fungi, either to gain nutrients, territory or as a defence response. The conceptual hypothesis is that competition and antagonism will promote or induce the expression of hydrolytic enzymes along with change in hyphal morphology as well as behaviour in interspecific interactions of fungi. In case of the deadlock interactions for different fungi, it was observed that the two competing species could not grow further after they came in contact with one another. We observed them till no further change in the hyphal morphology macroscopically could been seen because there are reports where some of the barrages initially observed disappeared, most likely as a consequence of the invading fungi overwhelming the physical defences of the weaker fungi (Boddy 2000). This study showed that the pigmentation in the point of contact increased gradually till 14 days. This could be because of the strong competition between the two species for nutrition and space. So, when the nutrient became limited and gradually finished, the fungi became unable to perform their metabolism any longer and due to which the pigmentation in the interaction zone came to a halt. Several authors have associated the changes in pigmentation during fungus–fungus interactions to the activity and changes in the levels of phenoloxidases (Boddy 2000; Iakovlev et al. 2004). The barrages formed during the interaction of *T. coccinea* – *T. versicolor* as well as between *T. coccinea – L. lactinea* were very prominently marked by the presence of a brown coloured demarcation line at the point of contact.

**Table 2.** Interaction of the isolates in all possible combinations

T. versicolor F1	T. coccinea F3	T. cubensis F8	P. lacomtei F5	P. pulmonarius F6	P. pulmonarius F15	P. ostreatus F14	G. lucidum F2	G. gibbosum F16	L. lactinea F9	C. rosea F13	F. fujikoroi F10	F. oxysporum F11	F. incarnatum F12	
1	4	4	4	2	2	2	4	2	4	4	3	2	2	***T. versicolor* F1**
	1	3	3	2	2	2	4	2	4	3	3	3	3	***T. coccinea* F3**
		1	3	3	3	3	4	4	4	3	4	4	4	***T. cubensis* F8**
			1	4	4	4	4	3	3	3	3	4	4	***P. lacomtei* F5**
				1	4	4	2	2	3	3	4	4	4	***P. pulmonarius* F6**
					1	4	2	2	3	3	4	3	4	***P. pulmonarius* F15**
						1	2	2	3	3	3	3	4	***P. ostreatus* F14**
							1	4	4	3	4	4	4	***G. lucidum* F2**
								1	4	3	4	4	3	***G. gibbosum* F16**
									1	3	3	3	3	***L. lactinea* F9**
										1	4	4	4	***C. rosea* F13**
											1	4	4	***F. fujikoroi* F10**
												1	4	***F. oxysporum* F11**
													1	***F. incarnatum* F12**

Most of the interspecific fungal–fungal interaction leads to morphological changes in the interacting fungi (Boddy and Hiscox 2016). These morphological changes which lead to change in physiological behaviour are observed due to microscopic and enzymatic alterations during interaction. The microscopic changes during the deadlock process have so far not been studied in details. In our study, we observed the microscopic changes in the hyphal pattern during the interaction by light and scanning electron microscopy, so as to gain a better understanding on the change in surface morphology at the zone of interaction. Microscopic examination at the points of contact during the *in-vitro* dual-culture conditions involving *T. coccinea, T. versicolor* and *L. lactinea* revealed very competitive interaction with unusual hyphal changes like coiling, excessive hyphal network and excessive sporulation in either or both the fungi. Some of these microscopic characteristics are similar to those as previously described by several authors (Ujor et al. 2012; Ijoma et al. 2019). The microscopic changes can be attributed to the change in metabolic profiles which is a consequence of the strong competition between the two interacting fungi. This is in accordance with the reports of other authors who mentioned that fungal–fungal interactions lead to production of metabolites such as alcohols, aldehydes, ketones, terpenes, aromatic compounds and reactive oxygen species (ROS) (Heilmann-Clausen and Boddy 2005; Peay et al. 2008; Hu et al. 2011) along with secretion of extracellular enzymes, *viz*. laccase, cellulase, xylanase and peroxidases (Silar 2005). The interaction causes activation of compounds which remains silent or produced in less quantity, thus helping in nutrient acquisition and defence mechanism. Moreover, the coiling of the hyphae of *T. coccinea* during interaction can be considered as an outcome of self-defence by its own antifungal toxins ((Imtiaj and Lee 2007; Arfi et al. 2013).

Cellulose, hemicelluloses and lignin are the major components of lignocellulosic biomass (Isikgor and Becer 2015). Polysaccharides and lignin form the basic component of recalcitrant plant secondary cell was complex. The lignocellulosic biomass can be effectively hydrolysed through enzymatic activity involving the conversion of cellulose into glucose, and hemicelluloses into xylose by cellulase and xylanase, respectively (Zhang et al. 2014). The activity of both xylanase and cellulase activity increased in the fungal isolate under study in dual cultures when compared to that of the mono culture. This is similar to studies conducted earlier on interactions of *Pleurotus flabellatus* – *Pleurotus eous, Pleurotus flabellatus* – *Phanerochaete chrysosporium, Hypholoma fasciculare* – *Phanerochaete velutina, Trametes versicolor* – *Bjerkandera adusta, Trametes versicolor* – *Trichoderma harzianum* where there was increased cellulase activity due to greater demand of nutrients (Hiscox et al. 2010; Fukasawa et al. 2011; Parani and Eyini 2011)). It can thus be said that the two competing fungal species in this study have been induced to secrete cellulase and xylanase in order to better utilise the nutrients, so as to meet the increasing energy requirement during the interaction. As a result of greater cellulase and xylanase activities, the reducing sugar content was enhanced in the interaction as compared to the monocultures. Ma et al. (2011) reported, that the interactions of *Auricularia polytricha* with *Irpex lacteus* led to the induction of cellulase and xylanase activities which improved the lignocellulose saccharification compared to enzymes of the monoculture. The increase in xylanase and cellulase activities are also a consequence of defence response induced by either one or both the fungi (Cano-Canchola et al. 2000). Besides xylanase and cellulase, chitinase is also an important hydrolytic enzyme responsible for degradation of components of fungal cell wall. Like cellulase and xylanase, the increase in chitinase activity during fungal-fungal interaction can also be related to defence response and better nutrient acquisition. Similar observations of higher chitinase activity during fungal-fungal interaction of *Botryotinia fuckeliana-Bionectria ochroleuca* and *Trichoderma* asperellum- *Rhizoctonia solani* has been reported by other authors (Viterbo et al. 2002; Mamarabadi et al. 2008). Increased chitinase activity has also been observed during fungal-fungal interactions among higher basidiomycetes such as *Fomitopsis pinicola-Hypholoma fasciculare, F. pinicola-Resinicium bicolour* and *F. pinicola-Coniophora arida*. The chitinase has been described to play roles in morphogenetic changes as well as facilitation of cell walls to permit the entry of toxic and anti-fungal metabolites by the competing species during fungal-fungal interaction (Lindahl and Finlay 2006). Dual culture can elicit production of some specific compounds that have antimicrobial and antioxidant activities produced as a consequence of the defence mechanism by one or both the fungi during the interaction process. In order to combat with those antimicrobial and antioxidant compounds, either or both the fungi may enhance the production of these important hydrolytic enzymes (Metreveli et al. 2017). These enzymes have great demand in various fields, such as detergents production, processing in pulp and paper industry, as well as textiles, food, and feed industries. Because of these hydrolytic enzymes, lignocelluloses can act as inexpensive resources for fermentable sugars and chemicals (Fatokun et al. 2016).

### Conclusions

Microscopic studies of *in-vitro* fungal interactions revealed hyphal coiling, deformation, dense hyphal network at the interaction zone. Along with the morphological changes, biochemical changes like the overproduction of cellulase and xylanase were revealed during the interaction process. For commercial production of cellulase and xylanase, this type of interaction can help in increasing the enzyme production.

## Data Availability

– The datasets used and/or analysed during the current study are available from the corresponding author on reasonable request.

## Declarations

**Ethics approval consent to participate -** No animal or human volunteers were used in this study.

**Consent to participate** – Not Applicable.

**Adherence to national and international regulations** – Not Applicable.

**Consent for publication** – Not Applicable.
